# A Robot-Assisted Framework for Rehabilitation Practices: Implementation and Experimental Results †

**DOI:** 10.3390/s23177652

**Published:** 2023-09-04

**Authors:** Giorgia Chiriatti, Luca Carbonari, Maria Gabriella Ceravolo, Elisa Andrenelli, Marzia Millevolte, Giacomo Palmieri

**Affiliations:** 1 Department of Industrial Engineering and Mathematical Sciences, Polytechnic University of Marche, 60131 Ancona, Italy; g.chiriatti@univpm.it (G.C.); g.palmieri@univpm.it (G.P.); 2 Department of Experimental and Clinical Medicine, Polytechnic University of Marche, 60131 Ancona, Italy; m.g.ceravolo@univpm.it (M.G.C.); e.andrenelli@univpm.it (E.A.); 3 Neurorehabilitation Clinic, Ancona University Hospital, 60131 Ancona, Italy; marzia.millevolte@ospedaliriuniti.marche.it

**Keywords:** collaborative robotics, rehabilitation, human–robot interaction, interaction perception

## Abstract

One of the most interesting characteristics of collaborative robots is their ability to be used in close cooperation scenarios. In industry, this facilitates the implementation of human-in-loop workflows. However, this feature can also be exploited in different fields, such as healthcare. In this paper, a rehabilitation framework for the upper limbs of neurological patients is presented, consisting of a collaborative robot that helps users perform three-dimensional trajectories. Such a practice is aimed at improving the coordination of patients by guiding their motions in a preferred direction. We present the mechatronic setup, along with a preliminary experimental set of results from 19 volunteers (patients and control subjects) who provided positive feedback on the training experience (52% of the subjects would return and 44% enjoyed performing the exercise). Patients were able to execute the exercise, with a maximum deviation from the trajectory of 16 mm. The muscular effort required was limited, with average maximum forces recorded at around 50 N.

## 1. Introduction

The use of collaborative robots (cobots) in industry is gaining traction due to their great flexibility. Their success is mostly determined by the possibility of implementing workflows in a shared environment where both humans and robots can cooperate safely [1,2,3]. This feature is being exploited in application fields that are different from traditional production, where the use of classical industrial robots would be impossible due to obvious safety reasons. Healthcare, for example, is a widely investigated field that many researchers have focused on since it provides a wide range of challenges and applications [4,5,6].

For years, the robotics field has played a pivotal role in providing assistance, and the use of collaborative robots is not a novelty [7]. Yin and Yushenko [8], among other authors, used a collaborative robot for surgeries; Colucci et al. [9] developed a mobile service robot exploiting a Kinova lightweight cobot; Mišeikis et al. [10] tested an assistive mobile collaborative manipulator prototype in retirement facilities. Researchers have been further driven by the COVID-19 pandemic [11], which made the lack of healthcare operators even more evident, and stressed the importance of human–human distancing in certain environments (e.g., hospitals and geriatric yards). Many applications involve rehabilitation. For example, Kajopoulos et al. [12] designed a robot-assisted training protocol based on joint attention responses for children with autism. Kyrkjebø et al. [13] analyzed the potential of the UR5, a collaborative robot from Universal Robots, for the rehabilitation of stroke patients. Gherman et al. [14] envisaged the use of a cobot for upper limb rehabilitation, while Prendergast et al. [15] focused on shoulder diseases. For further details on the use of cobotics in the rehabilitation scenario, see [16,17,18,19,20,21].

This paper follows up on the analyses conducted over the last two years, as detailed in references [22,23,24]. We propose a mechatronic implementation of a platform for rehabilitation practices. The testbench was developed to specifically enhance the ability of stroke patients to follow specific trajectories with their upper limbs. Such directions have been constrained via a collaborative robot (namely, a UR5e by Universal Robots), using a novel control law described subsequently. The collaborative robot is able to help the user perform expected motions by preventing different directions using a dedicated impedance control law. It is worth noting that, from a clinical point of view, regarding the neurorehabilitation of patients, there would be no advantage in an *assisted mode* where the robot guides the patient’s hand through a given trajectory. The main purpose of the exercise is not to simply increase the joint range of motion, or strengthen the upper limb muscles, but to promote the relearning of goal-directed activities (such as picking up objects from a table). This outcome can only be achieved by allowing patients to produce an active movement, thus engaging the complex neural network underlying the generation of task-oriented gestures. The target of the motion can be dynamically moved by the exercise supervisor in order to optimize the result. For such reasons, no assisted modalities were implemented that involved the robot dragging the patient’s limbs.

In the easiest version of the exercise, the robot applies weak force in the same direction applied by the patient. However, the control on the handle trajectory is completely on the patient’s ability to perform the motion, while the robot simply provides minimum help, barely enough to overcome the machine’s internal friction.

The novelty of the method lies in the reconfigurability of the exercise based on the patient’s needs and the physician’s expertise. In contrast to other approaches, the parameters of the exercise can be set almost completely, satisfying the particular needs of each patient. From a technological point of view, an additional advantage involves the use of commercial hardware that can offer flexibility, reliability, technical and maintenance support, and relatively low costs. In fact, collaborative robots are becoming increasingly common in industrial plants nowadays, and costs are decreasing, just as commercial and technical support is increasing. Finally, the cobots also offer facilitated programming tools that allow even inexperienced operators to create programs of some complexity in a short time, promoting flexibility and personalization of therapy.

**Figure 1:**
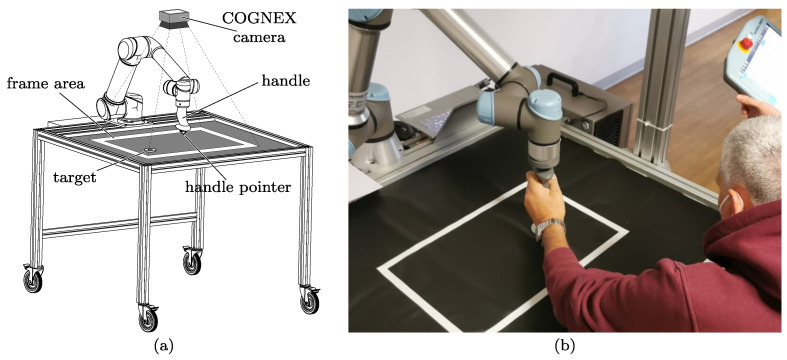
(**a**) CAD model of the rehabilitation device; (**b**) patient exercising during a preliminary experimental test.

The rest of the paper describes the rehabilitation device in detail (Figure 1) and presents a series of experimental data collected in preliminary clinical trials on neurological patients.

## 2. Rehabilitation Framework Description

As mentioned, the main objective of the rehabilitation framework is to train the ability of stroke patients to follow a given trajectory chosen by the physiotherapist to accomplish a task. Thus, UR5e, a collaborative robot by Universal Robots, has been equipped with a handle that is specifically designed for individuals who, due to mild to moderate spasticity, struggle to exert grasping force or have difficulty opening their hands. The patients are asked to move the handle, which is provided with a pointer, toward an object, which serves as a target. The position is dynamically recorded by a COGNEX camera and transmitted to the UR5e controller via TCP communication. A specific program was developed to make the robot accomplish two basic tasks: helping the patient in running the linear trajectory toward the target, and hindering possible deviations from that path by means of an elastic pull-back force. The implementation was conducted using Polyscope 5, by Universal Robots software, Odense, Denmark. For patient safety, robot velocity and accelerations were limited; moreover, the framework allowed patients to sit outside of the robot’s reach to prevent accidental head contact.

### 2.1. Exercise Phases

**Figure 2:**
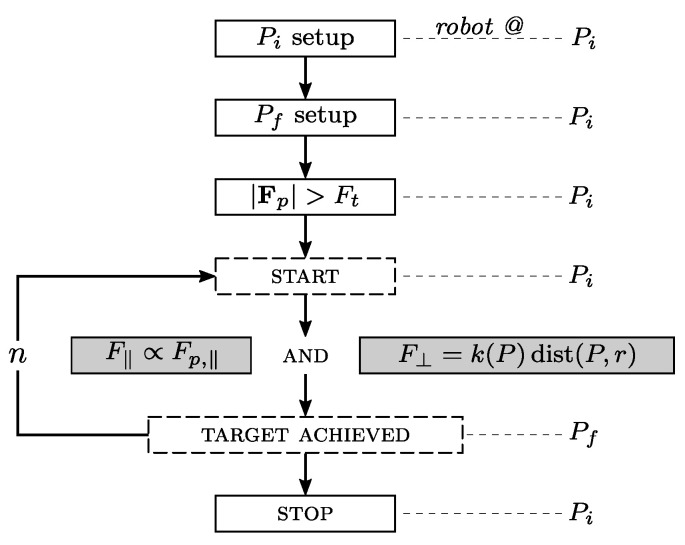
Main phases of the rehabilitation exercise.

**Figure 3:**
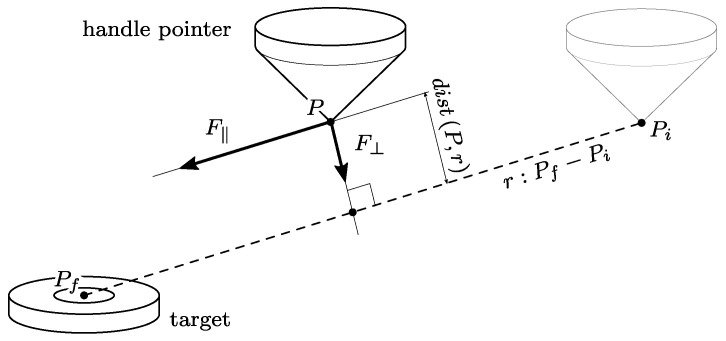
Force components of the UR5e robot thrust.

The exercise phases are shown in Figure 2. Briefly, they can be described as follows:

1. The caregiver (or patient, if he/she is able) sets the starting point $P_{i}$ of the trajectory by positioning the handle (i.e., the UR5e end-effector, *EE*) in front of the subject, so that it can be grabbed comfortably.
2. The caregiver chooses a final point $P_{f}$ for the trajectory by moving the ring target on the plane of the bench. The COGNEX camera frames the target’s coordinates and communicates them to the robot.
3. The EE is steadily maintained at $P_{i}$ until the patient exerts force $F_{p}$ that is greater in the module than a pre-defined threshold value (possibly customized to the patient’s characteristics) $F_{t}$:
  (1) $$\left|F_{p}\right|>F_{t}$$
  From that point, the actual exercise begins, and the robot acts according to a control law, which will be described later in the text.
4. During the exercise, which is cycled for a number *n* of times, and decided a priori, the robot exerts force, which depends on the patient’s interactions and the EE tip point position (called *P*), with respect to line $r:P_{f}-P_{i}$. In particular, two components of force are of interest, as described in Figure 3: a component $F_{\|}$ that is parallel to line *r* and proportional to the force, exerted by the user in the same direction ($F_{p,\|}$ is measured by means of the onboard force sensor), and a component $F_{⊥}$ that is parallel–perpendicular to *r*, configured as a variable stiffness elastic force.
5. The exercise repetition is considered complete when the EE tip reaches the target position $P_{f}$. When this occurs, the cobot moves the handle to $P_{i}$, driving back the patient’s hand to the starting point.
6. Once the subject completes *n* repetitions, the exercise is over, and the patient’s hand is moved back to $P_{i}$; the caregiver then decides if a further set of repetitions has to be conducted with identical or new force parameters.

### 2.2. Control Law

As mentioned above, the three components of force to be exerted by the cobot are computed to pursue two different aims: to help the motion along line *r*, and to contrast any deviation from such a trajectory. Thus, two components of the human–robot interaction force are considered, i.e., parallel and perpendicular to *r* (see Figure 3).

Component $F_{\|}$ was chosen to be proportional to the component of the force applied by the patient to the handle ($F_{p}$) parallel to *r*, denoted here as $F_{p,\|}$. To provide the exercise with a further degree of customization, the proportionality can be selected by the caregiver, according to three different intensity levels:

- **easy**$\to F_{\|}=cF_{p,\|}$: The robot applies force toward the target, actively helping the patient in reaching the target. The constant *c* is set to 0.5, following the suggestions of professional caregivers who personally tested the device.
- **medium**$\to F_{\|}=0$: The robot does not apply force toward the direction of the target. Therefore, the patient must apply the necessary force to move the handle, as if the robot is set in *free-drive* in that direction.
- **difficult**$\to F_{\|}=-cF_{p,\|}$: The robot counteracts the patient by providing force toward the opposite direction of the desired motion.

It is worth noting that a proper set of safety protocols and force thresholds was implemented to prevent the robot from pushing on the patient’s hand in an uncontrolled manner. These programming details are not specified here for the sake of conciseness, and are complementary to the standard safety settings of collaborative robots.

**Figure 4:**
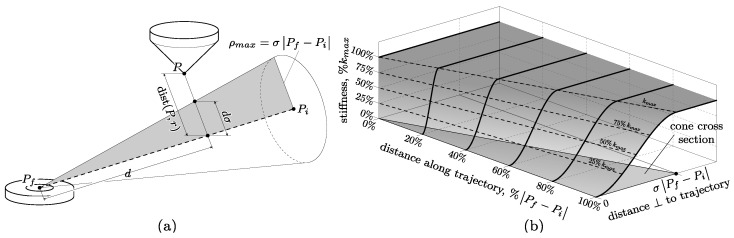
(**a**) Conic transition space around the line *r*; (**b**) trend of the stiffness used for impedance control of the UR cobot.

The force component that is orthogonal to the ideal direction of motion is determined by a variable stiffness law designed to provide smooth robot reactions, especially across the *r* line. In addition, according to the suggestions of practitioners, it is useful for patients to have higher compliance when the handle pointer is far from the target, while it should become stiffer when approaching $P_{f}$. To achieve this purpose, a variable stiffness *k* is implemented, as graphically presented in Figure 4. In a few words, a conic transition space is defined around line *r* (Figure 4a). Within such a space, the stiffness follows a cubic trend, from 0 (when P∈r, i.e., when the patient follows the trajectory) to a maximum value $k_{max}$ on the surface of the cone (as shown in Figure 4b). The apex of the cone coincides with $P_{f}$, while its aperture is given by the maximum radius $\rho_{max}$ reached at $P_{i}$. For simplicity, $\rho_{max}$ is set proportionally to the distance $\left|P_{f}-P_{i}\right|$. Here, the constant σ that rules the proportionality is σ=1/3. Thus, the cone radius is a linear function of the distance *d* between the handle pointer *P* and the target $P_{f}$ projected on the line *r*, i.e., ρ=σd.

The formula is as follows:

(2) $$k:\left\{\begin{array}{ccc} \mathrm{dist}(P,r)\leq d\sigma & \Rightarrow & k^{*}\\ \mathrm{dist}(P,r)>d\sigma & \Rightarrow & k_{max} \end{array}\right.$$

where *d* is the distance between the handle pointer *P* and the target $P_{f}$ projected on the line *r*, as shown in Figure 4a; the stiffness $k^{*}$ is a cubic function of dist(P,r), expressed by

(3) $$k^{*}=\frac{k_{max}\mathrm{dist}{\left(P,r\right)}^{2}}{{\left(d\sigma \right)}^{2}}\left(3-2\;\frac{\mathrm{dist}(P,r)}{d\sigma }\right)$$

The maximum stiffness $k_{max}$ is set at 20 N/mm. It should be noted that the robot’s maximum force is quite limited (50 N), providing an intrinsic force saturation, which ensures the overall safety of the application.

## 3. Experimental Results

**Figure 5:**
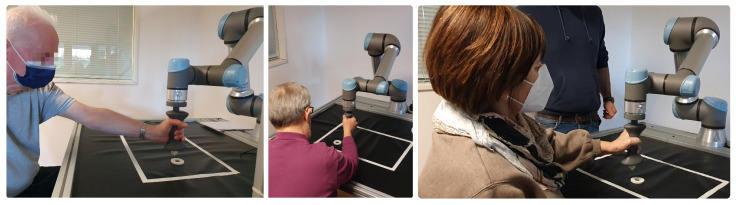
Participants performing the experimental session.

Ten patients (mean age of 66.90 years ± 6.93) with chronic stroke (>6 months) attending the outpatient neurorehabilitation clinic of Marche Polytechnic University in Ancona were enrolled. In addition, a control group of nine healthy subjects (mean age 33 years ± 5.20) participated in a single experimental session. Figure 5 shows some participants performing the exercise. Patients were enrolled if they presented a disabling upper limb paresis (as proxied by a muscle strength score of 2 to 4 on the Medical Research Council test at the shoulder, elbow, and wrist/finger levels) and were free from moderate to severe upper limb spasticity (i.e., if they exhibited a modified Ashworth Scale score of less than 2 at any level). Exclusion criteria for both patients and controls included severe cognitive deficits, pain, disabling comorbidities, drug or alcohol abuse, or any conditions that would prevent them from providing informed consent.

**Figure 6:**
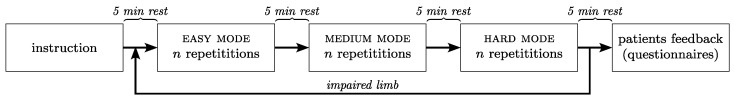
Timeline of the study protocol.

The timeline of the study protocol is shown in Figure 6. At the beginning of the experimental session, all subjects were informed about the goal of the exercise and how to interact with the robot. After a short break, each subject had to independently perform the exercise. The patient group was asked to perform three repetitions of the exercise (i.e., repeating the passages three times from phase three to five, as enumerated in the previous section), while the control group was asked to perform the exercise five times. In this way, each patient was not overloaded during the therapy session. At the end of the session, all participants were given a qualitative questionnaire to gauge their acceptance of the exercise. None of the subjects found the exercise difficult to perform (except for patient no. 1, who failed to complete it); moreover, they were not afraid of robotic technology. Furthermore, 52% of the subjects would return for another therapy session; however, the exercise should be modified to enhance stimulation and motivation, as after several trials, some subjects began to feel bored. However, the system was well accepted by volunteers and medical staff. Only two subjects declared that they preferred human interaction rather than sitting in front of a machine to perform therapy. Additional comments emerged about the handle system, especially from the patient group; some found it difficult to see the pointer under the handle and, consequently, had a hard time reaching the center of the target.

The participants completed the protocol without adverse events related to the device. Only subject no. 1 was unable to complete the exercise and was not considered in the data analysis. Table 1 presents the demographic characteristics of all participants. In the $P_{i}$ setup phase, the caregiver asked each volunteer to grasp the handle with their arm relaxed for 5 s and then apply high force to the handle. This phase was necessary to personalize the exercise and establish the force limits (maximum and minimum) of the training in order for it to be accomplished effortlessly.

**Figure 7:**
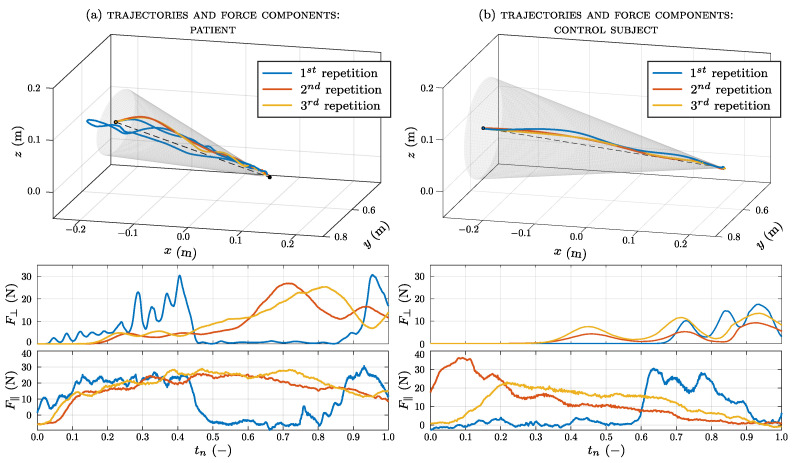
Example of the experimental results in terms of trajectories and force components for a patient (**a**) and a control group subject (**b**).

The average of the maximum force measured in the setup phase of the patient group is 43.45 (21.98) N; for the control group, it is 50.2 (33) N. The executed trajectories and forces recorded by the robot are shown in Figure 7a for one patient and in Figure 7b for one healthy subject. These figures are related to an execution in easy mode, which is the first modality tested. The trajectory created by the subject’s hand during the exercise to reach the target is derived from the pose coordinates of the robot. In order to make the three repetitions comparable, the force components are plotted versus a normalized time abscissa. The actual times for the three executions were 26.82 s, 5.4 s, and 6.04 s (mean 12.75 s) for the patient and 8.35 s, 4.13 s, and 3.99 s (mean 5.49 s) for the healthy subject. As can be seen, deviations from the line $P_{f}-P_{i}$ are larger for the patient, with respect to the control subject. In particular, during the first repetition, the patient does not follow a linear movement but deviates from the planned trajectory. This may be due to the fact that during the first trial, the patient does not yet have confidence in the system. Moreover, the deviations at the beginning of the exercise (next to point $P_{i}$), are higher, likely due to the force threshold that the subject is asked to overcome in order to begin the motion (phase 3 of the exercise). Despite such deviations, the perpendicular (or radial) force $F_{⊥}$ in this region of space is mostly low since the end-effector is provided with great compliance. On the contrary, the robot strictly guides the subject’s hand next to $P_{f}$, where the transition between the null stiffness and $k_{max}$ is pretty fast. The parallel (or longitudinal) force $F_{\|}$ is an indicator of how much the robot is helping the subject in performing the exercise; for the three repetitions, its value is different for each subject. In general, the average value of absolute force required for the user to perform the exercise (which is 1/c times that exerted by the robot) is ∼6 N. It is worth noting that the rehabilitation value does not lie in the muscular effort, but in the coordination required to achieve the goal of planning and following a trajectory.

**Figure 8:**
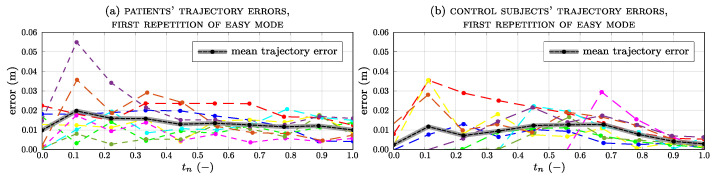
Trajectory errors for all subjects and average value for patients (**a**) and control subjects (**b**) during the first repetition in easy mode.

The trajectory’s error is defined as the difference between the real trajectory and the ideal one. The quantitative results from the trajectory analysis are listed in Table 2, which shows the mean error calculated for the impaired and the control group in each working mode (i.e., easy, medium, and hard). For comparable results between the two groups, errors are calculated based on trajectories performed by the impaired arm in the patient group and the non-dominant arm in the control group. The trajectory executed by the patient group deviates more from the planned one than that performed by the control group; the largest mean error for both groups lies in the easy mode, with an overall value of 0.016±0.01 m (for the patient case) and 0.008±0.004 m (for the control case). Even if patients have coordination problems, they still manage to perform the exercise with low error values. Figure 8 shows the trend of the trajectory error for each subject and the average error (in black) for all subjects during the first repetition of the easy modality (Figure 8a for patients, Figure 8b for the control group). In patients, the average error is the highest at the beginning of the exercise (20%), reaching a value of 0.02 m. In the control group, the error slightly increases from 30% and 50% phases and decreases from 70% in the completed exercise. However, the error is still very low and subjects are able to follow the planned trajectory smoothly.

**Figure 9:**
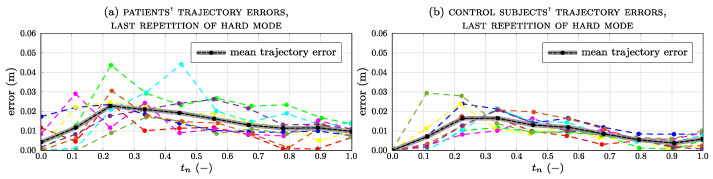
Trajectory errors for all subjects and average value for patients (**a**) and control subjects (**b**) during the last repetition in hard mode.

Figure 9 shows the trajectory error trend and the average for all subjects during the last repetition in the hard-working mode. As each subject performed the exercise several times, the average curves for both groups are similar; however, the errors for the patients are slightly higher than for the healthy subjects, peaking at around 30% of the exercise’s completion.

Force data recorded by the robot’s TCP are listed in Table 3 for the patient group and Table 4 for the control group, where the mean, minimum (min), and maximum (max) force values are reported with the standard deviations (std) in the three modes. The mean values in the control group increase as the difficulty of the exercise increases; however, for the patients, there is a lower value in the medium mode, while in the remaining modes, the values are approximately the same. This is because the differences between each mode are very small and the perception of increased difficulty is minimal. This strategy was devised with clinical staff to prevent muscle fatigue in patients. The minimum values, on the other hand, are proportional to the increase in difficulty, and the negative sign indicates its opposite direction, with respect to the direction of movement. This is an indicator that the subject is braking or decelerating the handle. Since patients apply more effort to complete the exercise, their maximum forces are higher than those in healthy subjects and, in both groups, the higher forces are in the hard mode.

**Figure 10:**
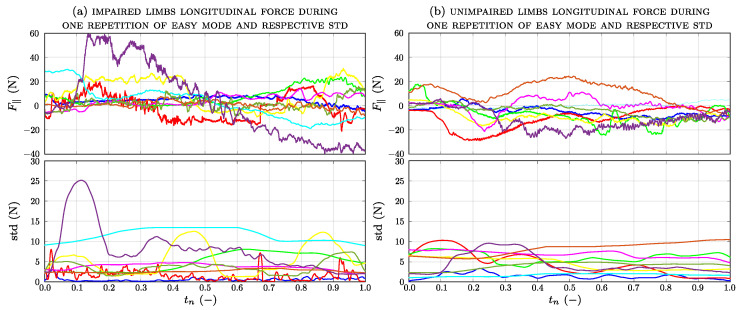
Longitudinal force during one repetition of the easy mode of all patients: (**a**) impaired and (**b**) unimpaired limbs.

**Figure 11:**
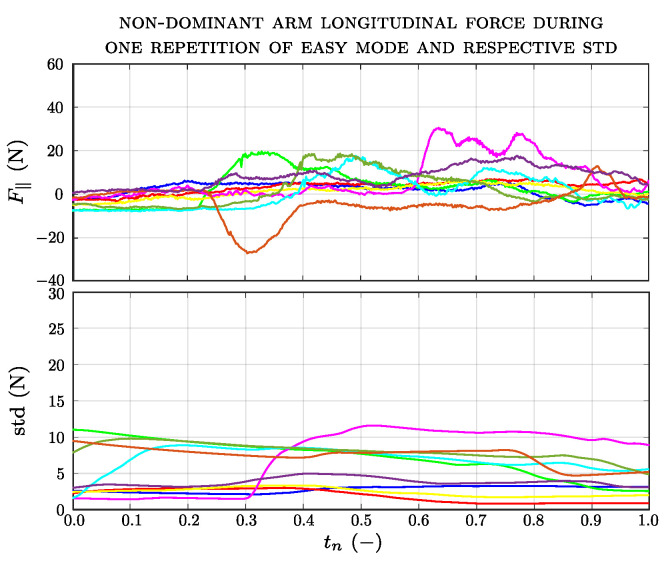
Longitudinal force during one repetition of the easy mode of all participants of the control group.

Figure 10a shows the longitudinal force applied by each impaired limb and the corresponding standard deviation calculated over a sliding window of 500 samples. Only the longitudinal force applied by patient no.10 differs significantly from the general trend of all the others, ranging between −20 N and 30 N. Figure 10b shows the longitudinal force and the sliding standard deviation of each unimpaired limb applied by the patient group. In most cases, the values of the forces are similar to those applied by the impaired limb, while their trends are different. In the case of the impaired arm, the trend of the standard deviation presents higher peaks. Figure 11 shows the longitudinal force applied by the non-dominant arm and the corresponding standard deviation for the control group.

## 4. Discussion

The authors would like to point out that the primary focus of this manuscript is not on the clinical benefits of an extended rehabilitation process; this research is aimed at providing the preliminary impressions of patients and physicians regarding the rehabilitation process. For this reason, the number of participants and collected results were limited to better appreciate some technical information on the mechatronic framework. A metric for evaluating the efficacy of the robot-assisted framework from a clinical point of view should come after its repeated and structured use. In practice, it would be interesting to analyze the long-term effects on patients to understand how (and how much) the approach is beneficial for the neurorehabilitation of patients. However, the purpose of the paper is foundational with respect to such considerations; therefore, the quality metric for movement is only represented by the error between the anticipated linear trajectory and the trajectory actually performed by the patients.

We should also note that the framework is targeted at hemiparetic patients, aiming to train their capacity to follow simple trajectories (e.g., lines) toward a target without deviating from the shortest path. The exercise phases, which have been refined in collaboration with professional caregivers, were presented with the robot’s control law. This law was developed to assist motion along the linear trajectory and counteract any deviation from it. This task was accomplished by using force proportional to that exerted by patients along the trajectory, with an elastic pull-back in the perpendicular direction.

The development of the framework allowed us to perform experimental tests on 9 healthy volunteers and 10 chronic stroke patients. The participants completed the protocol without adverse events related to the device. The three modes developed with the exercise were designed to produce a force that helps (or counteracts) the training subject. The trajectory executed by the patient (Figure 7a) differs from the trajectory of the healthy subject (Figure 7b), especially for the first repetition. At the beginning of the exercise, the patients did not know the right direction to follow. Progressively, in subsequent repetitions, the patients became familiar with the exercise and discerned the right direction to follow. The average trajectory errors were always greater than those of healthy subjects. However, in both groups, the error reached its highest mean value in the easy modality, respectively, registering 0.016±0.01 m for the patient group and 0.008±0.004 m for the control group. After all repetitions, patients discerned the right direction to follow, leading to a reduction in trajectory errors.

The force data recorded by the robot’s end-effector show that the average baseline force in patients (43.45 (21.98) N) is lower than in healthy subjects (50.2 (33) N), as expected in subjects with upper limb paresis. During the exercise, patients strained their arms more and exerted higher force than healthy subjects, especially in the hard mode. Since the patients had more difficulty performing the exercise, they applied higher force to compensate for their arm weakness.

The robotic system was well-accepted by volunteers (52% of the subjects would return for another therapy session and 44% enjoyed the exercise) as well as the therapists who supervised the exercise.

The system enables the recording of therapy data, ranging from the tracking of the executed trajectory to the interaction force applied by each subject. The robot’s sensors evaluate how much help the patient needs, ensuring that the exercise is at the appropriate level required by the subject. Moreover, the therapy could be prolonged through intensive sessions requiring minimal intervention from the therapist, who has to supervise the exercise.

## 5. Conclusions

In this paper, a novel framework for robot-assisted rehabilitation practices is presented; the main objective is to use industrial commercial hardware that can offer safety, flexibility, and reliability, paving the way for large-scale implementation that benefits the increasing number of treated patients. In addition, the current programming tools offered by cobots allow for a high degree of therapy customization with little software reconfiguration time. The framework targets stroke patients with upper limb paresis, aiming to train their capacity to follow simple trajectories (e.g., lines) toward a target without deviating from the shortest path. To achieve this, a collaborative UR5e robot was equipped with a specifically designed handle. The exercise phases, which were refined in collaboration with professional caregivers, were presented alongside the robot’s control law. This law was developed to assist motion along the linear trajectory and counteract any deviation from it. The task was accomplished by using force proportional to that exerted by patients along the trajectory, and an elastic pull-back in the perpendicular direction. The development of the framework allowed us to conduct experimental tests on non-sick volunteers and neurological patients.

None of the subjects found the exercise difficult to perform (except for patient no. 1, who failed to complete it); moreover, they were not afraid of robotic technology. Furthermore, 52% of the subjects would return for another therapy session; however, the exercise should be modified enhance stimulation and motivation, as after several trials, some subjects began to feel bored. However, the system was well accepted by volunteers and medical staff. Only two subjects declared that they preferred human interaction rather than sitting in front of a machine to perform therapy. Additional comments emerged about the handle system, especially from the patient group; some found it difficult to see the pointer under the handle and, consequently, had a hard time reaching the center of the target.

Future research should focus on the suitability of this framework in training hemiparetic stroke patients, both in their subacute and chronic stages, to ensure repetitive, intensive, and engaging training of proper duration to stimulate brain plasticity, reduce motor impairment, and increase functional capacity [25,26,27,28].

## Figures and Tables

**Table 1:** Demographic characteristics of participants.

Category	Impaired Group	Control Group
Gender—Male, Female	9 M, 1 F	7 M, 2 F
Impaired Hand—Right, Left	2 R, 8 L	-
Age—years, mean (std)	66.9 (6.93)	31 (5.2)

**Table 2:** Mean error between the planned and measured trajectories for the patient group and the control group.

Mode	Patients Group	Control Group
Easy	0.016±0.010 m	0.008±0.004 m
Medium	0.012±0.004 m	0.007±0.001 m
Hard	0.012±0.004 m	0.008±0.002 m

**Table 3:** Mean, min, and max values of the applied force for the patient group.

Mode	Mean ± Std	Min ± Std	Max ± Std
Easy	7.68±7.33 N	−8.83±8.49 N	22.01±8.18 N
Medium	4.82±9.56 N	−11.38±7.78 N	20.18±11.26 N
Hard	7.08±9.42 N	−13.45±5.73 N	24.88±12.49 N

**Table 4:** Mean, min, and max values of the applied force for the control group.

Mode	Mean ± Std	Min ± Std	Max ± Std
Easy	5.19±4.17 N	−5.46±5.63 N	16.50±9.31 N
Medium	5.45±4.80 N	−6.46±4.75 N	16.46±10.59 N
Hard	8.46±6.43 N	−9.51±5.54 N	22.75±17.62 N

## Data Availability

All subjects gave their informed consent for inclusion before they participated in the study. The study was conducted in accordance with the Declaration of Helsinki, and the protocol was approved by the Ethics Committee of Marche Polytechnic University.

## References

[1] Galin R., Meshcheryakov R. (2019). Automation and robotics in the context of Industry 4.0: The shift to collaborative robots. IOP Conference Series: Materials Science and Engineering.

[2] Maddikunta P.K.R., Pham Q.V., Prabadevi B., Deepa N., Dev K., Gadekallu T.R., Ruby R., Liyanage M. (2021). Industry 5.0: A survey on enabling technologies and potential applications. J. Ind. Inf. Integr..

[3] Chiriatti G., Palmieri G., Scoccia C., Palpacelli M.C., Callegari M. (2021). Adaptive Obstacle Avoidance for a Class of Collaborative Robots. Machines.

[4] Kyrarini M., Lygerakis F., Rajavenkatanarayanan A., Sevastopoulos C., Nambiappan H.R., Chaitanya K.K., Babu A.R., Mathew J., Makedon F. (2021). A survey of robots in healthcare. Technologies.

[5] Holland J., Kingston L., McCarthy C., Armstrong E., O’Dwyer P., Merz F., McConnell M. (2021). Service robots in the healthcare sector. Robotics.

[6] Abbas M., Narayan J., Dwivedy S.K. (2023). A systematic review on cooperative dual-arm manipulators: Modeling, planning, control, and vision strategies. Int. J. Intell. Robot. Appl..

[7] Fareh R., Elsabe A., Baziyad M., Kawser T., Brahmi B., Rahman M.H. (2023). Will Your Next Therapist Be a Robot? A Review of the Advancements in Robotic Upper Extremity Rehabilitation. Sensors.

[8] Yin S., Yuschenko A. (2019). Collaborative robot-surgeon assistant. Extrem. Robot..

[9] Colucci G., Tagliavini L., Carbonari L., Cavallone P., Botta A., Quaglia G. (2021). Paquitop. arm, a Mobile Manipulator for Assessing Emerging Challenges in the COVID-19 Pandemic Scenario. Robotics.

[10] Mišeikis J., Caroni P., Duchamp P., Gasser A., Marko R., Mišeikienė N., Zwilling F., De Castelbajac C., Eicher L., Früh M. (2020). Lio-a personal robot assistant for human-robot interaction and care applications. IEEE Robot. Autom. Lett..

[11] Yang G.Z., Nelson B.J., Murphy R.R., Choset H., Christensen H., Collins S.H., Dario P., Goldberg K., Ikuta K., Jacobstein N. (2020). Combating COVID-19—The role of robotics in managing public health and infectious diseases. Sci. Robot..

[12] Kajopoulos J., Wong A.H.Y., Yuen A.W.C., Dung T.A., Kee T.Y., Wykowska A. (2015). Robot-assisted training of joint attention skills in children diagnosed with autism. Social Robotics, Proceedings of the 7th International Conference, ICSR 2015, Paris, France, 26–30 October 2015.

[13] Kyrkjebø E., Laastad M.J., Stavdahl Ø. Feasibility of the UR5 industrial robot for robotic rehabilitation of the upper limbs after stroke. Proceedings of the 2018 IEEE/RSJ International Conference on Intelligent Robots and Systems (IROS).

[14] Gherman B., Alin B., Jucan D., Fidelian B., Carbone G., Pisla D. (2019). Upper limb rehabilitation with a collaborative robot. Acta Tech. Napoc.-Ser. Appl. Math. Mech. Eng..

[15] Prendergast J.M., Balvert S., Driessen T., Seth A., Peternel L. (2021). Biomechanics Aware Collaborative Robot System for Delivery of Safe Physical Therapy in Shoulder Rehabilitation. IEEE Robot. Autom. Lett..

[16] Yuan F., Klavon E., Liu Z., Lopez R.P., Zhao X. (2021). A systematic review of robotic rehabilitation for cognitive training. Front. Robot. AI.

[17] Giansanti D. (2021). The social robot in rehabilitation and assistance: What is the future?. Healthcare.

[18] Dong M., Zhou Y., Li J., Rong X., Fan W., Zhou X., Kong Y. (2021). State of the art in parallel ankle rehabilitation robot: A systematic review. J. Neuroeng. Rehabil..

[19] Qin L., Ji H., Chen M., Wang K. (2023). A Self-Coordinating Controller with Balance-Guiding Ability for Lower-Limb Rehabilitation Exoskeleton Robot. Sensors.

[20] Peñaloza Gonzalez J.A., Gonzalez-Mejia S., Garcia-Melo J.I. (2023). Development of a Control Strategy in an Isokinetic Device for Physical Rehabilitation. Sensors.

[21] Wolański W., Michnik R., Suchoń S., Burkacki M., Chrzan M., Zadoń H., Szaflik P., Szefler-Derela J., Wasiuk-Zowada D. (2023). Analysis of the Possibility of Using the UR10e Cobot in Neurological Treatment. Actuators.

[22] Chiriatti G., Palmieri G., Palpacelli M.C. (2020). A framework for the study of human-robot collaboration in rehabilitation practices. International Conference on Robotics in Alpe-Adria Danube Region.

[23] Chiriatti G., Carbonari L., Costa D., Palmieri G., Niola V., Gasparetto A., Quaglia G., Carbone G. (2022). Implementation of a Robot Assisted Framework for Rehabilitation Practices. Proceedings of the Advances in Italian Mechanism Science.

[24] Chiriatti G., Bottiglione A., Palmieri G. (2022). Manipulability Optimization of a Rehabilitative Collaborative Robotic System. Machines.

[25] Straudi S., Baluardo L., Arienti C., Bozzolan M., Lazzarini S.G., Agostini M., Aprile I., Paci M., Casanova E., Marino D. (2022). Effectiveness of robot-assisted arm therapy in stroke rehabilitation: An overview of systematic reviews. NeuroRehabilitation.

[26] Duret C., Grosmaire A.G., Pila O., Breuckmann P. (2022). Robot-assisted therapy for upper limb paresis after stroke: Use of robotic algorithms in advanced practice. NeuroRehabilitation.

[27] Everard G., Declerck L., Detrembleur C., Leonard S., Bower G., Dehem S., Lejeune T. (2022). New technologies promoting active upper limb rehabilitation after stroke: An overview and network meta-analysis. EuropEan J. Phys. Rehabil. MEdicinE.

[28] Zhang L., Jia G., Ma J., Wang S., Cheng L. (2022). Short and long-term effects of robot-assisted therapy on upper limb motor function and activity of daily living in patients post-stroke: A meta-analysis of randomized controlled trials. J. Neuroeng. Rehabil..

